# Variance and Autocorrelation of the Spontaneous Slow Brain Activity

**DOI:** 10.1371/journal.pone.0038131

**Published:** 2012-05-30

**Authors:** Yoshiki Kaneoke, Tomohiro Donishi, Jun Iwatani, Satoshi Ukai, Kazuhiro Shinosaki, Masaki Terada

**Affiliations:** 1 Department of System Neurophysiology, Graduate School of Wakayama Medical University, Wakayama, Japan; 2 Department of Neuropsychiatry, Graduate School of Wakayama Medical University, Wakayama, Japan; 3 Wakayama-Minami Radiology Clinic, Wakayama, Japan; Bellvitge Biomedical Research Institute-IDIBELL, Spain

## Abstract

Slow (<0.1 Hz) oscillatory activity in the human brain, as measured by functional magnetic imaging, has been used to identify neural networks and their dysfunction in specific brain diseases. Its intrinsic properties may also be useful to investigate brain functions. We investigated the two functional maps: variance and first order autocorrelation coefficient (***r*** **_1_**). These two maps had distinct spatial distributions and the values were significantly different among the subdivisions of the precuneus and posterior cingulate cortex that were identified in functional connectivity (FC) studies. The results reinforce the functional segregation of these subdivisions and indicate that the intrinsic properties of the slow brain activity have physiological relevance. Further, we propose a sample size (degree of freedom) correction when assessing the statistical significance of FC strength with ***r*** **_1_** values, which enables a better understanding of the network changes related to various brain diseases.

## Introduction

Spontaneous fluctuations of blood oxygen level-dependent (BOLD) signals, as measured by functional magnetic resonance imaging (fMRI), are not simply caused by random noise but represent brain functions. Functional connectivity (FC) analysis [1] of these signals, pioneered by Biswal et al. [2], has revealed various brain networks that are related to specific functions [3]–[5] and their relationship with brain diseases [6], [7]. The investigation of intrinsic properties of spontaneous BOLD fluctuations also revealed the other aspects of the brain function. Garrett et al. showed the relationship between standard deviation of BOLD signals and chronological age [8] and Baria et al. showed distinct spatial distributions of BOLD signals that reflect regional functional complexity [9]. These studies suggest that the intrinsic properties of BOLD signals provide novel information about regional differentiation of the brain.

In this study, we measured the intrinsic properties of spontaneous BOLD fluctuations using the two parameters, variance and autocorrelation coefficient, both of which were extracted by the autocorrelation function. The autocorrelation function of a time series is the correlation between its past and present states; thus, a high correlation indicates that the series state does not so change over time. The autocorrelation function has been used to extract periodicity in unitary neuronal activity [10]–[12] because conventional frequency analysis such as fast Fourier transform cannot be directly applied. Application of the autocorrelation function to continuous time series data such as BOLD signals is useful in extracting two distinct properties, i.e., variance and the first-order autocorrelation coefficient (***r*** **_1_**). The autocorrelation function is usually shown after normalization so that the correlation at various lags is between −1 and 1 (that is, the correlation coefficient). The value at lag zero before normalization divided by the sample number of the time series corresponds to the variance of the data. The value of ***r*** **_1_** can be calculated by the correlation at lag zero and lag 1. We show here that the spatial distributions of these values are different from each other and the distributions have functional relevance. Further, ***r*** **_1_** is useful for the correction of the degree of freedom during the assessment of FC strength between the data from two time series.

## Methods

### Participants

Twenty eight healthy subjects (13 women and 15 men, mean age: 34.5+/−7.3 years) were recruited for this study. All were right-handed according to the Edinburgh Handedness Inventory [13] with a mean score of 92+/−10.5 and gave informed consent prior to the study. This study protocol was approved by the Ethics Committee of Wakayama Medical University and we obtained written informed consent from all participants involved in this study.

### MRI Data Acquisition

Each subject’s brain structural and resting state functional images were acquired on a 3 Tesla MRI (PHILIPS, The Netherlands) using a 32-channel head coil (SENSE-Head-32CH). High-resolution three-dimensional T1-weighted anatomical images were collected with the following parameters: TR = 7 ms, TE = 3.3 ms, FOV = 220 mm, Matrix scan = 256, slice thickness = 0.9 mm, flip angle = 10°. Functional data were acquired using a gradient-echo echo-planar pulse sequence sensitive to BOLD contrast [14] with the following parameters: TR = 3000 ms, TE = 30 ms, FOV = 192 mm, Matrix scan = 64, slice thickness = 3 mm, flip angle = 80°. Three runs, each of which comprised 107 volumes (for 5 min 21 s), were administered to each subject. During acquisition, the subjects were instructed to stay awake with their eyes closed.

**Figure 1:**
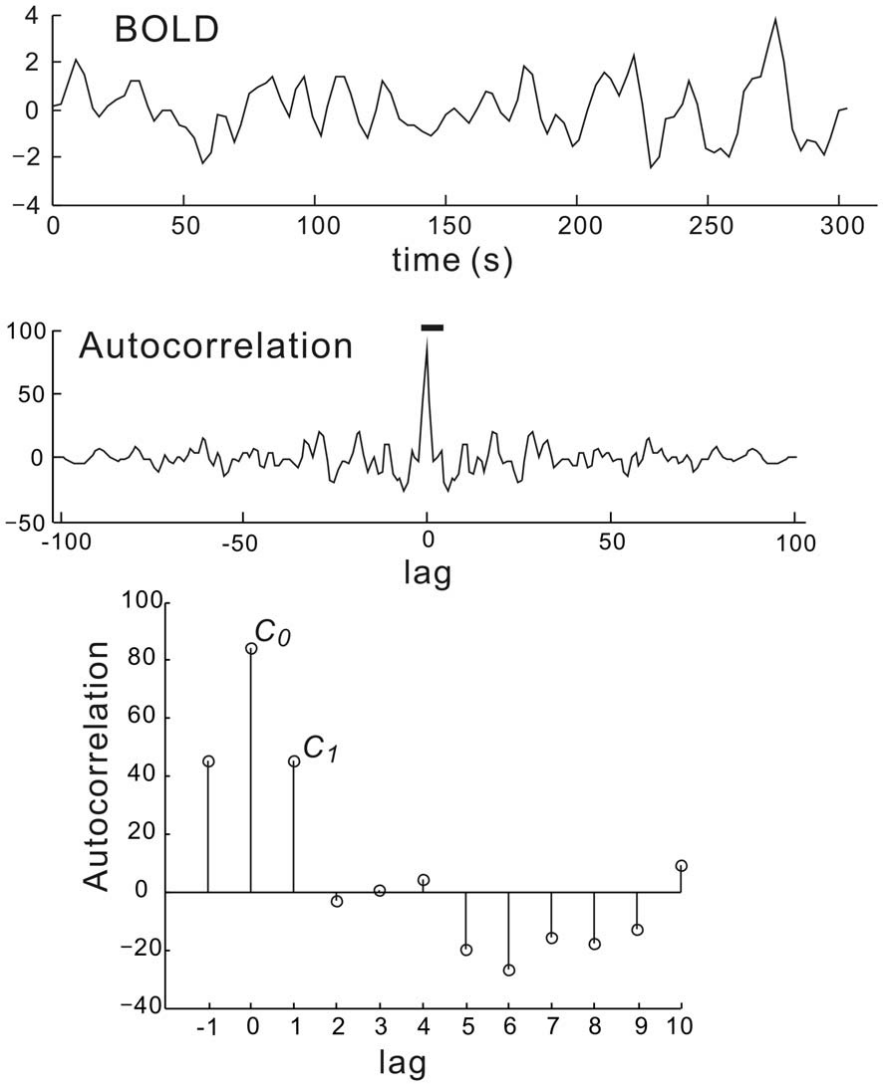
Autocorrelation function of BOLD signals for 306 s. The bottom graph shows the data for the lag range from −1 to 10, which is indicated by the bar in the middle graph. Note that the autocorrelation is not normalized.

### MRI Data Analysis

Preprocessing of functional MRI (fMRI) data was conducted using SPM8 (http://www.fil.ion.ucl.ac.uk/spm) and in-house software developed with MATLAB (Mathworks, Natick, MA, USA). The first 5 volumes of each fMRI acquisition run were discarded to allow for T1-equilibration effects leaving 102 consecutive volumes per session. Rigid body translation and rotation were used to correct head motion, and spatial normalization was achieved by 12-parameter affine transformation to the International Consortium for Brain Mapping Echo-Planar Imaging template in SPM8. Each image was resampled to 2-mm isotropic voxels and spatially smoothed using an 8-mm full width at half maximum Gaussian kernel. Similarly normalized and resampled structural images were then used to extract time series data for the cerebrospinal fluid (CSF), white matter (WM), and gray matter (GM),which were used to reduce non-physiological noise in the time series of BOLD signals (see below). Each subject’s three tissue images (CSF, WM, GM) were generated using SPM8 with a probability threshold of 90%.

**Figure 2:**
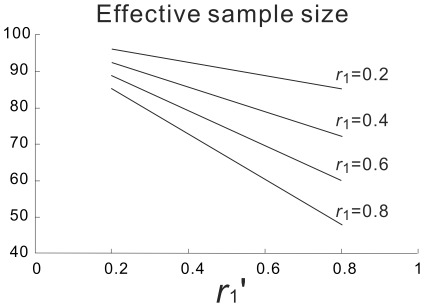
Effective sample size calculated with various *r*_1_ and *r*_1_’ values. The original sample size is 102. The effective sample size decreases as the autocorrelation coefficient decreases.

Exclusion of the signals unrelated to brain function (i.e., brain tissue fluctuations due to head motion, cardiac activity, and respiration) was done using CompCor [4], [15] and global signal regression [16]. Briefly, CompCor includes the following steps: identification of voxels showing the highest temporal variation (top 2%), principal component analysis (PCA) of these voxels and voxels within CSF and WM, identification of the PCA components accounting for a significant proportion of the variance in the data, and exclusion of the identified signal time course for each voxel using linear regression. Temporal (band-pass) filtering (ranging from 0.01 to 0.1 Hz) removed constant offsets and linear trends over each run. The 102 preprocessed images from each session were concatenated into single four-dimensional (time and 3 spatial data) images and, thus, the data from 3 sessions for each subject were used for the following analysis.

**Figure 3:**
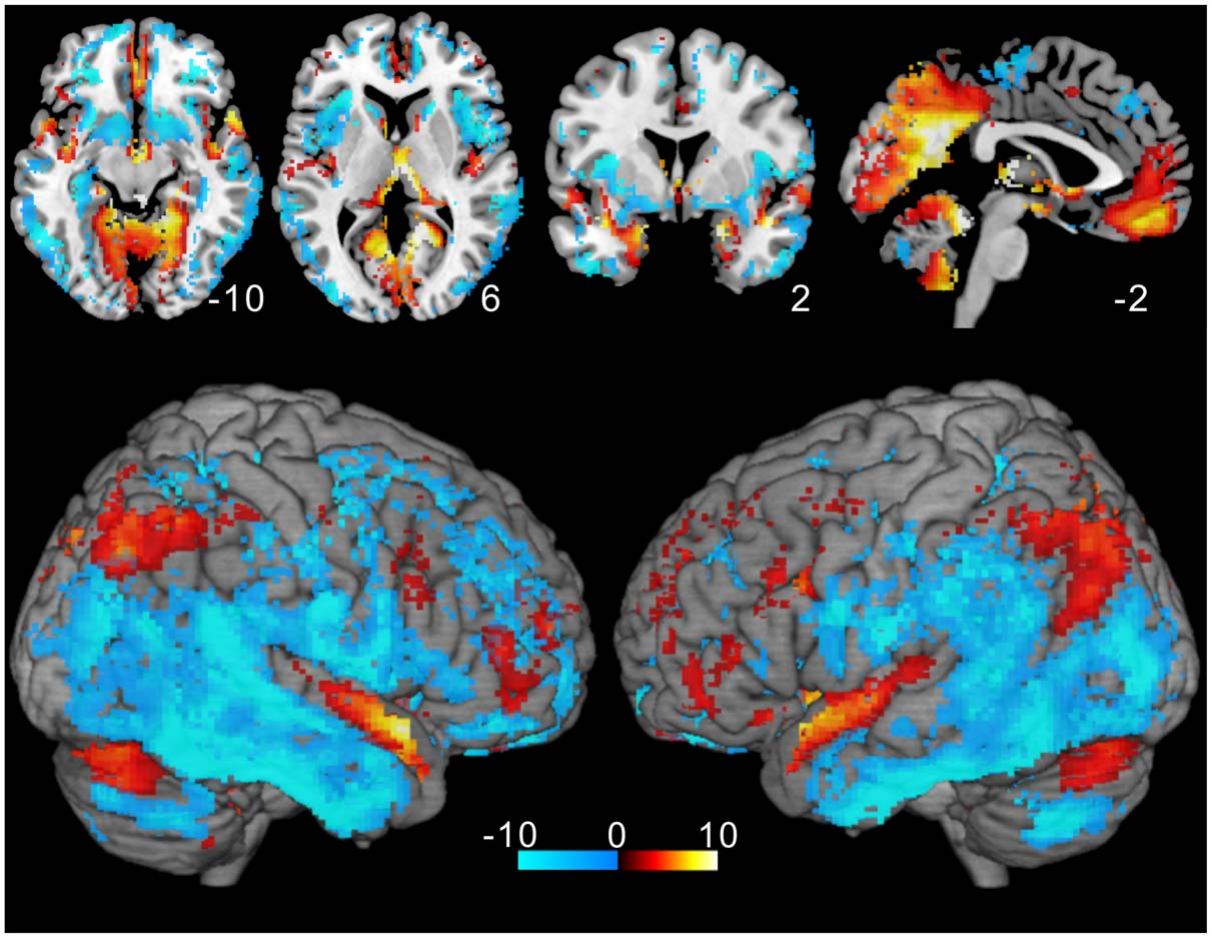
Variance *t*-value map. The result of one-sample ***t***-test is shown excluding non-significant voxels (p>0.05 with FDR corrected). High variance is seen in restricted cortical regions, such as the vmPFC, insula, PCC, calcarine sulcus, and lateral parietal lobes.

**Table 1:** High variance cortical regions: Brodmann’s area (BA); Z-score (Z, mean (SD)).

Region	MNI			BA	Z
	x	y	z		
Left superior temporalgyrus	−34	10	−29	20	2.54(1.29)
Left precuneus	−3	−60	32	31	2.02(1.16)
Right superior temporalgyrus	34	10	−29	20	1.83(1.13)
Left parahippocampus	−17	0	−25	28	1.78(0.92)
Left calcarine fissure andsurrounding cortex	−3	−84	5	17	1.68(1.96)
Left lingual gyrus	−12	−55	1	18	1.65(1.03)
Left rectus	−4	50	−19	11	1.57(1.06)
Right calcarine fissure andsurrounding cortex	4	−83	6	17	1.55(1.63)
Right precuneus	4	−65	30	31	1.48(1.06)
Right lingual gyrus	12	−55	3	18	1.47(0.76)
Right parahippocampus	19	−1	−23	28	1.24(0.97)
Right angular gyrus	47	−66	49	39	1.11(2.14)
Left angular gyrus	−48	−62	50	39	1.01(1.6)

The autocorrelation function (not normalized by dividing the values at lag 0, which is also known as autocovariance function) for each voxel of the functional GM volumes was calculated with the custom Matlab command (**Fig. 1**) and the variance (***v***) and first-order (lag 1) autocorrelation coefficient (***r*** **_1_**) were calculated for each voxel using the following equations:
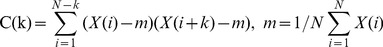
(1)

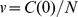
(2)

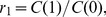
(3)where C(k) is autocorrelation at lag *k* of *N* sample data (*x*_1_, *x*_2_, …, *x*_N_) (*N* = 102, in this study).

**Figure 4:**
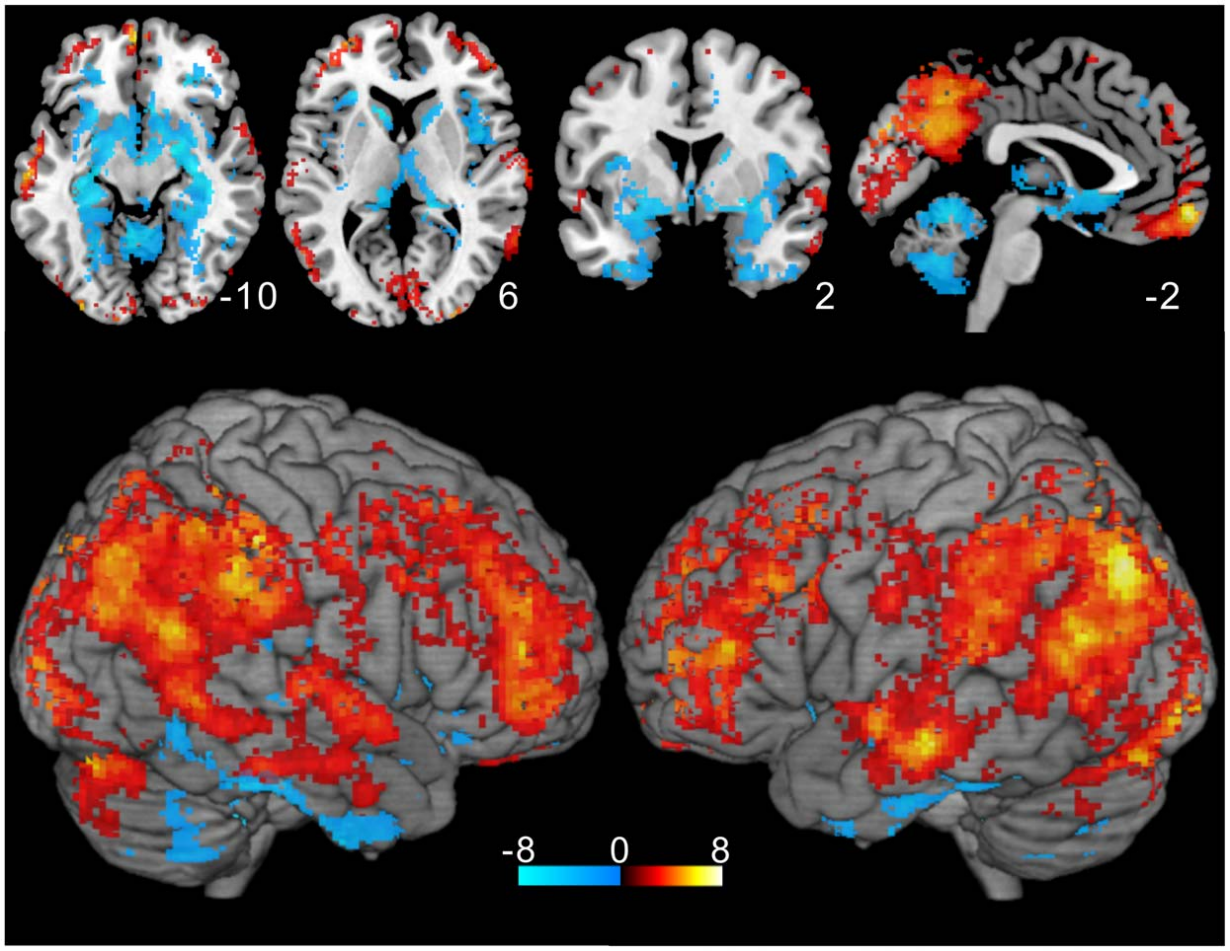
*r*_1_ *t*-value map. The result of one-sample ***t***-test is shown excluding non-significant voxels (p>0.05 with FDR corrected). Low ***r*** **_1_** values distributed around the caudal brain regions. Relatively high values are seen in the DMN regions and the cerebral cortex had the highest values except the insula and primary sensorimotor areas.

**Table 2:** High ***r*** **_1_** cortical regions: Brodmann’s area (BA); Z-score (Z, mean (SD)).

Region	MNI			BA	Z
	x	y	z		
Left PCC	−2	−59	31	31	1.2(0.6)
Left middle occipital gyrus	−41	−78	33	39	1.1(0.6)
Right supramarginal gyrus	65	−36	38	40	1.1(0.7)
Right angular gyrus	45	−74	36	39	1.0(0.9)
Left vmPFC	−4	58	−16	11	0.9(0.6)
Left inferior parietal cortex	−60	−45	41	40	0.9(0.7)
Left middle frontal gyrus	−41	49	4	46	0.7(0.7)
Right middle frontal gyrus	32	54	18	46	0.7(0.7)
Left middle temporal gyrus	−63	−16	−16	21	0.7(0.5)
Right superior temporal gyrus	63	−2	−6	21	0.7(0.7)
Right PCC	6	−55	31	31	0.5(0.6)
Right vmPFC	6	44	−22	11	0.5(0.9)
Left middle frontal gyrus	−42	28	36	45	0.5(0.7)

Then, the mean image for 3 sessions for each subject was calculated and standardized with the mean and standard deviation among all the voxels’ data. Each subject’s ***v*** and ***r***_1_ Z-score maps were used to extract voxels whose data were significantly different from mean zero, as assessed by a random effect one-sample *t*-test. The significance level was set at *p*<0.05 corrected for multiple comparisons with the false discovery rate (FDR) at 5% [17]. To assess the effect of lag, we created ***r*** **_2_** (lag 2) and ***r*** **_3_** (lag 3) maps and compared with ***r*** **_1_** map.

**Figure 5:**
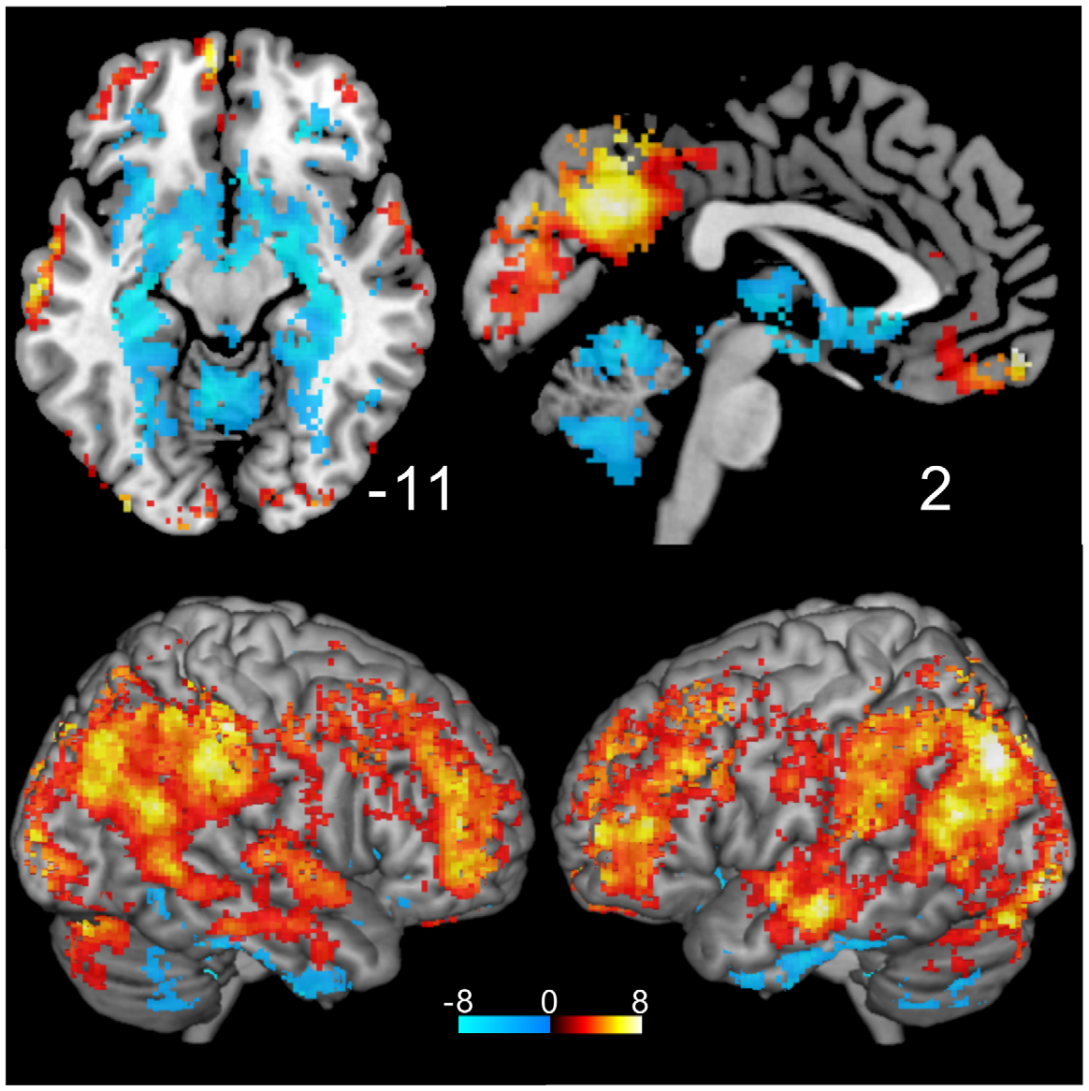
*r*_2_ t-value map. The result of one-sample ***t***-test is shown excluding non-significant voxels (p>0.05 with FDR corrected). The distribution pattern is similar to that for ***r*** **_1_** (Fig. 4).

The three dimensional presentations of the functional maps were created using MRIcron [18], which was also used to estimate the anatomical localization of the regions of interest. At each region of interest, we extracted the mean values of ***v*** and ***r_1_*** within a radius of 4 mm for each subject and assessed the regional difference of these values.

**Figure 6:**
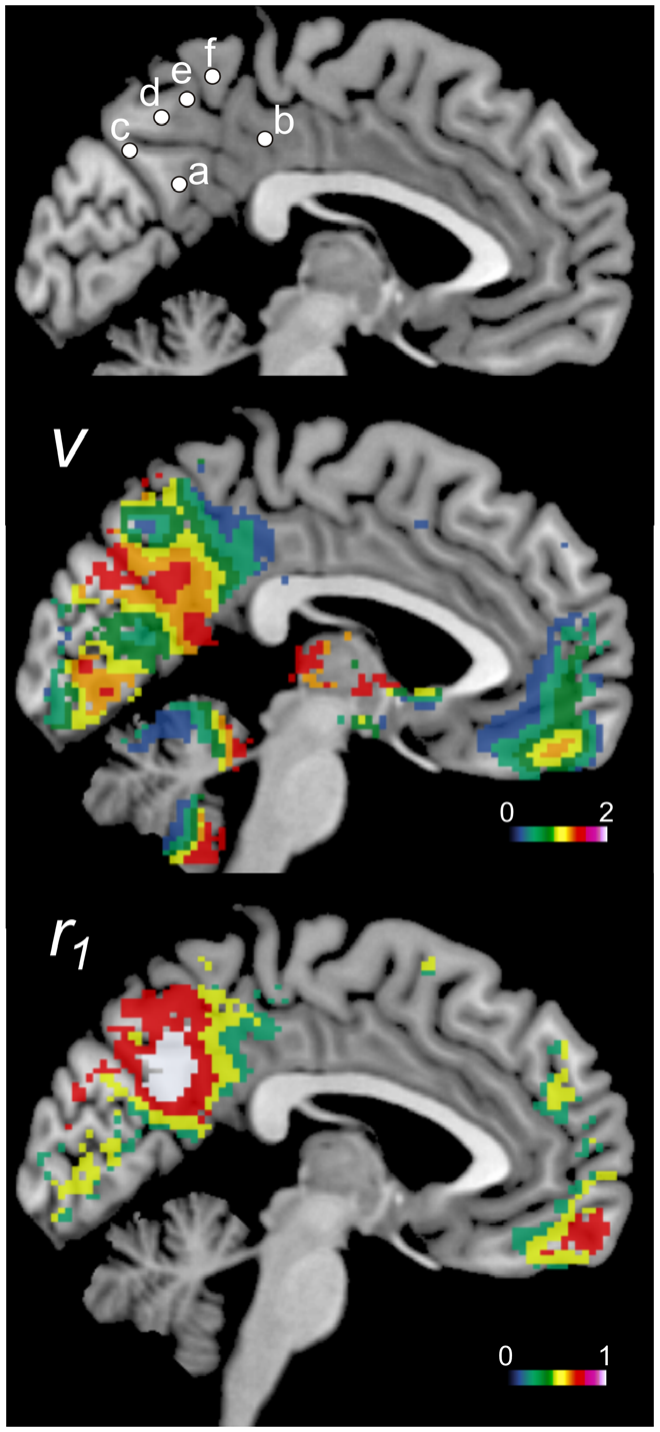
Relative locations of the seeds in the subdivisions of the precuneus and PCC shown with mean Z-score maps of *v* and *r*_1_. The mean Z-score map for the variance (***v***) is shown in the middle and the map for ***r*** **_1_** is shown in the bottom of the figure. Note that the distribution pattern for ***v*** is different from that for ***r*** **_1_** especially in the precuneus and PCC. **a**: ventral PCC; **b**: dorsal PCC; **c**: visual precueal region; **d**: cognitive/associative precuneal region; **e**: transitional zone; **f**: sensorimotor precuneal region.

**Table 3:** Seeded regions in the subdivisions of the PCC and precuneus.

Region	MNI		
	x	y	z
PCC			
Ventral part	2	−58	28
Dorsal part	2	−34	40
Precueus			
Sensorimotor region	−2	−47	58
Transitional zone	−2	−56	51
Cognitive/associative region	−2	−64	45
Visual region	−1	−75	36

**Figure 7:**
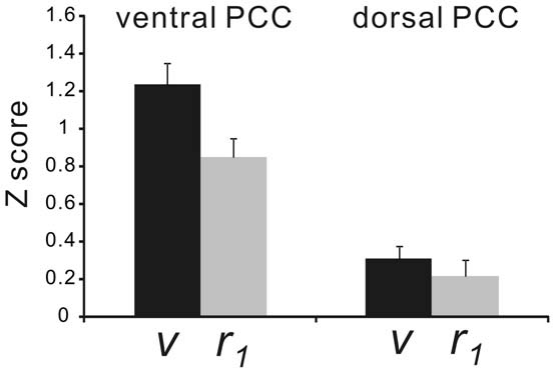
Z-scores of the variance (*v*) and *r*_1_ in the 2 subdivisions of the PCC. Both values were significantly higher in the ventral PCC than in the dorsal PCC (p<0.0001, paired ***t***-test).

### Estimation of Effective Sample Size by the Autocorrelation Function

The cross-correlation coefficient is commonly used to evaluate the magnitude of FC between two voxels in a functional volume [2], [19]. The statistical significance of the correlation between two *random* time series can be assessed by the *t* –test:
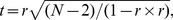
(4)where *r* denotes the correlation and *N* is the number of time points. Functional data, however, are not random [20], [21], i.e., the observed *N* samples are not independent from each other. Thus, the size of *N* has to be replaced by the *effective sample size* for correlation test, which is estimated from the following equation [22]:
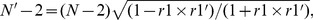
(5)where N′ is the effective sample size, and r1 and r1’ are the respective first order autocorrelation coefficients of the two time series. **Fig. 2** shows the effective sample sizes calculated using various values of r1 and r1’ when N = 102.

**Figure 8:**
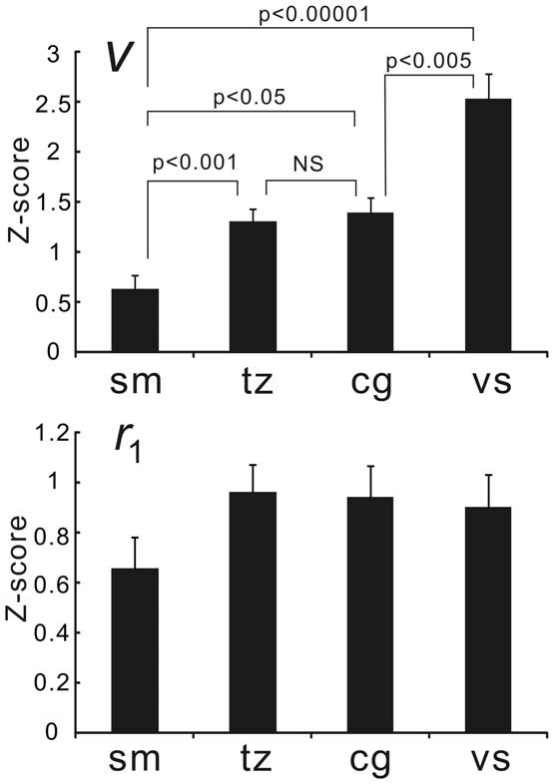
Z-scores of the variance (*v*) and *r*_1_ in the 4 subdivisions of the precuneus. Significant differences in the ***v*** values among the 4 regions were revealed by the paired ***t***-test (*p*-values were corrected with Bonferroni’s method). In contrast, there was no significant difference in the ***r*** **_1_** values among the 4 regions. Sm, sensorimotor region; tz, transitional zone; cg, cognitive/associative regions; vs, visual region.

We tested the effect of correcting the sample size on FC analysis. The default mode network (DMN) was assessed using the FC between the ventral part of the posterior cingulate cortex (PCC) (Montreal Neurological Institude (MNI) coordinates: 2, −58, 28) [23] and other brain regions. Pearson’s correlation coefficient between the ventral PCC and other brain regions was calculated for each subject. For the seed data, we used the mean time course of the voxels less than 4 mm from the center of the ventral PCC. We created 2 correlation image datasets; one was made with the values above the correlation threshold at *p*<0.05 when the degree of freedom was 100 (i.e., *N*−2). The other one was made with the values above the correlation threshold at *p*<0.05, but the degree of freedom was corrected using the two first order autocorrelation coefficients according to Equation (5).

**Figure 9:**
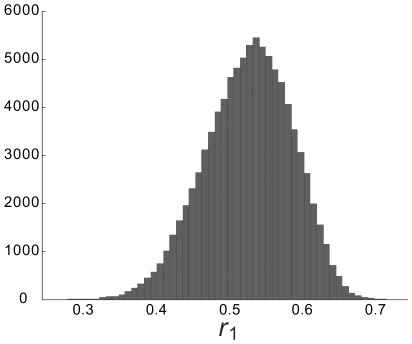
Distribution of the *r*_1_ values for the gray matter voxels in one subject. The value ranges from 0.3 to 0.7 though most of the data are between 0.5 and 0.6.

For the group analysis, setting a threshold at each subject data may not be appropriate because the data would not follow a Gaussian distribution even after Fisher’s ***r*** to ***z*** transform. Thus, we first converted the cross-correlation coefficient values to the ***t***-values using Equation (4) with *N’* for each voxel pair calculated by Equation (5). These ***t*** values were then converted to Z- scores with the same *N’* to perform one-sample ***t***-test as done in ***v*** and ***r***_1_. We used the ***t***-to-***z*** transform algorithm proposed by Hughett [24]. The result was compared with the Z-score map created with the conventional procedure that uses Fisher’s ***r*** to ***z*** transform.

**Figure 10:**
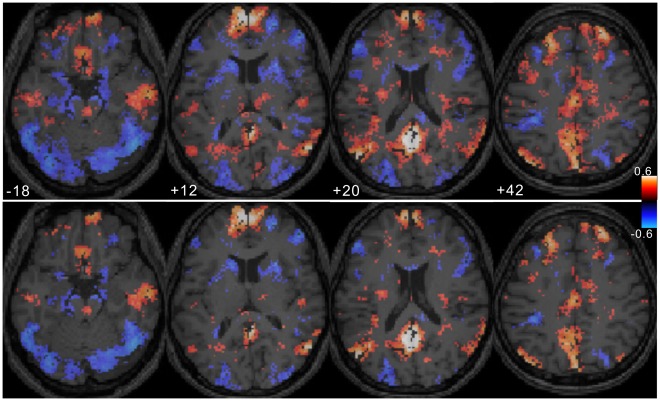
Effect of sample size correction. The top images show the distribution of the cross correlation coefficients (p<0.05, ***t***-test for each paired voxels’ data) between the ventral PCC and the other brain voxels without sample size correction (i.e., *N* = 102). The bottom images show the distribution of the voxels with the cross correlation coefficients that are significantly different from zero (p<0.05). The effective sample size (*N*’) (see text) was calculated for each pair of voxels with their autocorrelation coefficients and each pair’s *N*’ was used to assess the significance of the cross-correlation coefficient. For this subject, ∼46% of voxels were revealed not to be significant after sample size correction.

## Results

### Variance Map

**Fig. 3** shows the result of the one-sample ***t***-test for the variance as the ***t***-value map excluding non-significant voxels (p>0.05, FDR corrected). As seen, high variance of the spontaneous BOLD signals was found in the area of the PCC, precuneus, lateral parietal cortex, parahippocampus, superior temporal gyrus, and cerebellum. Notably, the precuneus, PCC, and medial occipital cortex were divided into several regions by the distribution of the variance. In the frontal lobe, the ventromedial prefrontal cortex (vmPFC) showed a higher level of variance than the other frontal areas. MNI coordinates and Z-scores for the high variance regions are shown in **Table 1**. In contrast, the insula, posterior temporal cortex, primary sensorimotor areas, and ventral striatum had low levels of variance (**Fig. 3**).

**Figure 11:**
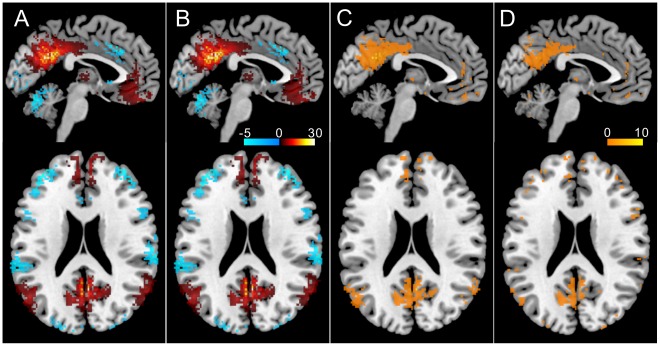
Functional connectivity strength seeding at vPCC. The results of one-sample ***t***-test are shown as ***t***-value maps excluding non-significant voxels (p<0.05 with FDR corrected) (**A**: the result for the conventional analysis; **B**: the result after the sampling size correction). Both patterns are nearly identical but the latter ***t***-values are higher than the former as shown by the difference map (**C**). The difference between the sample size corrected map and sample size uncorrected map created by the same ***r*** to ***t*** and ***t*** to ***z*** transform) is shown in D. The ***t***-values in the DMN regions for the sample size corrected map were higher than that for the uncorrected map though the Z-score distribution was the same (not shown).

### Autocorrelation Coefficient Map

**Fig. 4** shows the result of the one-sample ***t***-test for ***r_1_*** values as the ***t***-value map excluding non-significant voxels (p>0.05, FDR corrected). Cortical areas generally showed high ***r_1_*** values except the insula and primary sensorimotor area. Relatively high ***r_1_*** values were seen in the lateral parietal lobe, lateral prefrontal lobe, PCC, and vmPFC. Interestingly, these distributions correspond to the regions in which myelin developed last [25] and with the lowest myelin content [26]. Notably, the distribution of the ***r_1_*** values in the PCC and precuneus is different from the distribution of the variance. The MNI coordinates and Z-scores for the high ***r_1_*** regions are shown in **Table 2**. Significantly low ***r_1_*** values were observed in the amygdala, hippocampus, and cerebellum, in contrast to the distribution of the variance (see **Fig. 3**).

**Fig. 5** shows the result of the one-sample ***t***-test for ***r_2_*** values as the ***t***-value map excluding non-significant voxels (p>0.05, FDR corrected). As seen, the distribution pattern is nearly identical to the map for ***r_1_*** (see Fig. 4). No significant voxels were found for ***r_3_*** Z-score values.

### Posterior Cingulate Cortex and Precuneus

Recent studies showed that the PCC and precuneus have distinct FC properties and that these areas could be divided into several subdivisions. Because the ***v*** and ***r_1_*** maps showed significantly higher values in these regions, it is of interest to check if these two values are different among the proposed subdivisions of the PCC and precuneus. **Fig. 6** shows the mean Z-score maps across all subjects for both values excluding non-significant voxels (p>0.05, FDR corrected). On the basis of previous studies [23], [27], we seeded 2 and 4 locations in the PCC and precuneus, respectively. Their relative locations are shown in **Fig. 6** and the MNI coordinates are shown in **Table 3**. **Fig. 7** shows the mean (+/−SEM) ***v*** and ***r_1_*** Z-score values among the subjects for the PCC subdivisions. Remarkable differences in both values were seen between the two subdivisions. The ventral part of the PCC showed significantly higher ***v*** and ***r_1_*** values than the dorsal part PCC (*p*<0.0001, paired ***t***-test).

Three subdivisions in the precuneus showed significantly different variance from each other (**Fig. 8**). The sensorimotor region had the lowest variance, and while visual region had the highest. The variance of the cognitive region variance was not significantly different from that of the transitional zone. In contrast, these regions had similar ***r_1_*** values and the subdivisions could not be differentiated by ***r_1_***.

### Effective Sample Size

**Fig. 9** shows the distribution of ***r_1_*** for the gray matter voxels in one subject’s brain. The range from 0.3 to 0.7 indicates that the effective sample size for the assessment of the cross correlation coefficient could vary from 59 to 91 when the original sample size is 102 according to Equation (5).

The effect of sample size correction on the selection of significant (*p*<0.05) FC voxels is shown in **Fig. 10**. As seen, the area was much reduced and 46.1% of voxels in the whole brain were excluded by the correction of the sample size. The mean (+/−SD) percent of the excluded voxels across the subjects was 43.4+/−4.7%.

**Fig. 11** shows the results of one-sample ***t***-test for the Z-scores without (**A**) and with temporal autocorrelation correction (**B**) as the ***t***-maps excluding non-significant voxels (p>0.05, FDR corrected). The difference (corrected map minus conventional map that uses Fisher’s ***r*** to ***z*** transform) of these results is shown in **Fig. 11C**. Both procedures revealed the same distribution pattern but the ***t*** values were generally higher for the corrected map than the uncorrected map. Further, we checked the effect of sample size correction on the Z-score map by comparing the map created by the same procedure without sample size correction, that is the map was created by the same ***r*** to ***t*** and ***t*** to ***z*** transform using the original sample size (n = 102). The distribution of the uncorrected map was the same as that for the corrected map. **Fig. 11D** shows the difference between sample size corrected map and sample size uncorrected (otherwise the same as sample size corrected map) map. As seen, the ***t*** values in DMN regions were higher for the corrected map than the uncorrected map.

## Discussion

We created two human brain maps using the distribution of the variance (***v***) and ***r*** **_1_**, both of which were calculated from the autocorrelation function for each voxel. These maps showed distinct spatial distributions and had functional relevance because the precuneal and PCC subdivisions had significantly different values.

Although spontaneous fluctuations of BOLD signals has been clearly shown to have functional relevance in a number of FC studies, signal variance (or standard deviation) itself in a certain time period has not been paid much attention until recently [8], [28]. One possible reason is that BOLD signal variance could be largely affected by non-physiological noise such as heart beat, respiration, and head movements. Thus, proper preprocessing of BOLD time series data is the cardinal step for the assessment of variance as shown by Garrett et al. [8].

The physiological relevance of the higher variance seen in the superior temporal gyrus, lateral parietal lobe, vmPFC, cerebellum, and parahippocampus (**Fig. 3**) is not known. Variance is thought to be related to efficient neural processes [8], [29]. If its distribution was found to be related specifically to the resting state, the functional relationship of these regions to the DMN would be an interesting subject of a future study. One of the outstanding results in this study is that the subdivisions in the precuneus and PCC had significantly different distributions of the variance (**Figs. 7** and **8**). These subdivisions were proposed on the basis of previous FC studies [23], [27]. Our results indicate that these regions have distinct intrinsic activity that might be important for their specific functions using the related neural networks. Further, the unique distributions seen in the precuneus and PCC (**Fig. 6**) suggest that these regions could be divided into even more subdivisions, which was unexpected from the findings of previous studies [27], [30].

Previously proposed parameter, amplitude of low-frequency fluctuation (ALFF) [31], [32] would give the same results as the variance map when the same frequency range and proper standardization procedure was used. Detail analysis using ALFF and other frequency analysis may reveal further detail functional localizations in the precueus and PCC. We used variance instead of ALFF in this study simply because variance was lag zero autocovariance (see Methods).

The autocorrelation coefficient (***r*** **_1_**) distribution map revealed a distinct difference between the two subdivisions of the PCC (**Fig.** **6**). Namely, the ventral part of the PCC had higher ***r*** **_1_** and ***v*** values than the dorsal part, which provides further support for the fractionation of the PCC in addition to its distinct cytoarchitectonics [33] and differential activation and FC during a cognitive task [23]. The results indicate that future DMN studies should take these subdivisions of the PCC into account. The distribution of ***r*** **_1_** in the precuneus and PCC was quite different from that of ***v*** (**Fig. 6**), which suggests that these values reflect distinct properties of the neural activity in the same region.

Interestingly, the map showing the high ***r*** **_1_** regions (**Fig. 4**) has some resemblance to DMN regions [7], low frequency power distribution [9], [31], [34], cortical hubs [6], low myelination regions [25], [26], and amyloid beta deposition in Alzheimer’s disease [35], [36]. Similarities between low frequency distribution and DMN [31], between cortical hubs and amyloid beta distribution [6], and between amyloid beta deposition and low myelination regions [35] have been reported. It is reasonable that the distribution of ***r*** **_1_** reflects low frequency power distribution rather than high frequency distribution because the phase is larger for lower frequency oscillations than for higher frequency oscillations. Although the physiological relevance of the other similarities is unknown, the clinical application of ***r*** **_1_** maps may become useful for the diagnosis of Alzheimer’s disease.

In most biological time series data, the first-order autocorrelation coefficient is the highest because the present value must be affected by the most recent past value if any relationship between past and present value exists. Indeed, using lags other than 1 was not successful because the distribution pattern for lag 2 was similar to that for lag 1 (**Fig. 5**). The analysis using lag 3 did not show any significant voxels probably because most of the coefficient values at lag 3 were not significantly different from the values for random process. The results also validate the procedure for sampling size correction using only first-order autocorrelation coefficient.

Sampling size (or degree of freedom) correction for the statistical assessment of FC strength is important for the time series with short acquisition time and short TR [19]. Further, the temporal correlation of BOLD signals that affects the degree of freedom can vary between subjects even in the same region, which must be taken into account when an FC study is carried out with different subject groups. This is because the difference in FC between the two groups could be due to the different temporal correlations. Temporal correlation is related to the intrinsic activity in a region but FC represents a cross-correlation between different regions. Thus, these measures detect distinct brain functions. We showed that the effective sample size estimated by each voxel’s ***r*** **_1_** value was useful to exclude ∼43% voxels, which were determined to have a significantly high correlation with the seed voxel before sample size correction. This sample size correction may also be useful to compare FC maps with different sample sizes.

The fact that the regions involved in DMN showed relatively high ***r*** **_1_** values (**Fig. 4**) raises a doubt about its robust functional connectivity, because functional connectivity strength is exaggerated by the high local ***r*** **_1_** values. We found it was not the case for DMN. The connectivity pattern was the same and the strength could be higher even after the sample size correction (**Fig. 11**). Thus, high temporal autocorrelation process in each DMN region might be caused by strong functional connectivity in the network. The usefulness of sample size correction was shown by the difference between sample size corrected map and uncorrected map (**Fig. 11D**). Even though each subject’s Z-scores in the DMN regions should have been reduced by the sample size correction, group analysis shows that *t*-values were higher for the corrected map than the uncorrected map. This indicates that the variance of FC strength among subjects was reduced by the sample size correction and suggests that sample size correction increases the possibility to detect physiologically meaningful FC.

In conclusion, we showed that the distinct distribution patterns of the two parameters, ***v*** and ***r*** **_1_**. Detailed human brain mapping with these parameters might be useful for the identification of the estimated 150–200 cortical areas [26], because these maps seem to represent distinct brain properties that have not been shown by other parameters. Further, we proposed a sample size correction method with ***r*** **_1_**, which will be important for future FC studies on brain diseases.
